# The Impacts of Asparagus Extract Fractions on Growth and Fumonisins Biosynthesis in *Fusarium Proliferatum*

**DOI:** 10.3390/toxins12020095

**Published:** 2020-01-30

**Authors:** Natalia Witaszak, Justyna Lalak-Kańczugowska, Agnieszka Waśkiewicz, Łukasz Stępień

**Affiliations:** 1 Department of Pathogen Genetics and Plant Resistance, Institute of Plant Genetics, Polish Academy of Sciences, Strzeszyńska 34, 60-479 Poznań, Poland; nwit@igr.poznan.pl (N.W.); lste@igr.poznan.pl (Ł.S.); 2 Department of Chemistry, Poznan University of Life Sciences, Wojska Polskiego 75, 60-637 Poznań, Poland; agnieszka.waskiewicz@up.poznan.pl

**Keywords:** asparagus, *Fusarium proliferatum*, fumonisins, plant-pathogen interaction, qPCR, UPLC/TQD

## Abstract

Asparagus is a genus consisting of over two hundred species of perennial plants. *Fusarium proliferatum* is a major asparagus pathogen and it biosynthesizes a variety of mycotoxins, of which fumonisins B are prevalent. Our previous studies on *F. proliferatum* strains indicated that asparagus extract affects the expression of *FUM1* gene, encoding polyketide synthase, a key enzyme of the *FUM* gene cluster governing the biosynthesis of fumonisins. An asparagus-derived *F. proliferatum* strain increased fumonisin B_1_ production after extract fractions’ addition, reaching the maximum 2 or 24 h after treatment. The cultures yielded between 40 and 520 mg of dry weight of mycelia after 14 days of cultivation. The differences in fungal biomass amounts between the whole extract and its fractions may result from synergistic effect of all bioactive compounds present in asparagus extract. Among extract fractions, the methanolic fraction had the highest effect on the dry weight of the mycelium reaching about a 13-fold increase compared to the control. Furthermore, we measured the relative expression of the *FUM1* gene. Due to the possible antifungal activity of tested extract fractions, future research will be focused on the identification of the *Asparagus officinalis* L. compounds responsible for this activity.

## 1. Introduction

Asparagus (*Asparagus* sp.) is a genus of perennial plants, consisting of over two hundred species (according to World Checklist of Selected Plant Families). This savory vegetable is a plant of high nutritional and pharmaceutical values but is also often used as an ornamental plant. Asparagus is a rich source of vitamins and minerals as well as antioxidants and saponins, which occur mainly in peels [1,2]. Thanks to saponins, asparagus has antifungal, antitumor, antidiabetic and hypolipidemic properties [3,4,5,6,7,8,9]. In traditional medicine, asparagus has been applied as a diuretic and laxative agent [2].

Asparagus occurs in the areas of Europe, Africa, Asia and Americas. Multiple applications make it an economically important crop plant and, thus, extensive knowledge about asparagus pathogens and diseases is necessary to effectively prevent the yield loss. Asparagus rust, purple spot as well as Fusarium crown and root rot are the most important diseases caused by *Puccinia asparagi, Pleospora herbarium* as well as *Fusarium proliferatum* and *Fusarium oxysporum* f. sp. *asparagi*, respectively [10,11].

*Fusarium* is a group of the most common plant pathogens occurring all over the world and damaging crop yield. *Fusarium proliferatum* is a very important representative among *Fusarium* genus and the species has the ability to infect a wide range of host-plants, including asparagus, maize, garlic, wheat, pea, pineapple, banana and many more [12]. It is mainly transferred and spread by seeds and crop residues. *F. proliferatum* biosynthesizes multiple mycotoxins, such as: beauvericin, moniliformin, fusaric acid and highly toxic fumonisins from group B. Moreover, this pathogen can survive in many ecological niches, but the optimal environment is a warm and humid climate as well as loam soils with pH of about 5 [13]. Such characteristics may indicate a high plasticity and excellent adaptability of *F. proliferatum* to environmental challenges.

Infection caused by *F. proliferatum* is manifested by yellowing, stunting and wilting of the organs of infected plants and is correlated with asparagus decline [13,14]. Management of *Fusarium* infections is very difficult because no effective fungicides against *Fusarium* are available and multi-stage actions including plant and soil protection as well as inoculum reduction are necessary [11,13]. Moreover, accumulation of *Fusarium* secondary metabolites in plants’ tissue and their possible harmful effects on human and animal health is an additional issue in effective plant protection [12].

Fumonisin B_1_ is among the most dangerous secondary metabolites biosynthesized by *F. proliferatum* strains in apical cells of hyphae [15]. FB_1_ synthesis is governed by the *FUM* gene cluster, which is localized on *F. proliferatum* chromosome 1. This mycotoxin is harmful to other microorganisms as well as plants, animals and human. The FB_1_ mechanism of action bases on the disruption of the sphingolipid biosynthesis, particularly sphingosine and sphinganine. These compounds are the precursors of sphingolipids—the basic phospholipids of cell membranes [16]. Chemical structure of FB_1_ is similar to sphingosine, thus, both molecules compete for the active site of the ceramide synthase. The enzyme is responsible for the acylation of sphingosine and sphinganine, which results in the formation of ceramides. Lack of activity of this enzyme causes the inhibition of sphingolipid biosynthesis, which is the reason for their deficiency in cell membranes [17,18]. Disruption of sphingolipid metabolism and associated fat peroxidation results in the increase of reactive oxygen species (ROS), damaging DNA and proteins [19,20]. In turn, ROS play an important role as signal transducers in many molecular processes, such as hypersensitivity response in plants. These processes are a part of plant immunity response called FB_1_-induced cell death, which is similar to hypersensitivity response (HR), a type of programmed cell death that protects the plant from further infection [21].

Relatively much is known about changes caused in the plant during *Fusarium* infection. First, a hypersensitivity response occurs, causing cell death of the infected area. Some reports indicate that fumonisin B_1_ is a virulence factor causing FB_1_-induced cell death, mediated by reactive oxygen species (ROS) activation, phytoalexins accumulation and pathogenesis genes overexpression [21,22]. Along with the pathogen’s attack, a systemic acquired resistance (SAR) is activated. In asparagus plants, *F. proliferatum* affects salicylic acid production, which is the main signaling molecule connected with systemic response [23]. The mechanism of this process is not fully known but may be similar to the other *Fusarium* representative—*F. graminearum*—which contains salicylic acid degrading enzymes [24,25].

Plant-pathogen interaction is a complex network of connections, actions and reactions. To fully understand its rules, it is necessary to examine in detail the changes observed for both organisms during the infection process. So far, researchers were focused on the impact of the pathogen on plant gene expression and metabolism. On the contrary, the effect of plant metabolites with antifungal activity like camalexin, pisatin and resveratrol were less studied and fungal genes and biochemical pathways associated with the response against plant effectors are still not well-researched and understood [26,27,28]. In the case of *Fusarium*, reports on mechanisms occurring in the plant’s organism are available, but limited information has been gathered about molecular mechanisms of the reaction of *Fusarium* under biotic stress conditions. Therefore, it is necessary to continuously improve the knowledge about them, particularly for economically important crops.

Our previous studies on *F. proliferatum* strains indicated that asparagus extract affects the expression of *FUM1* gene, encoding a key enzyme of the *FUM* gene cluster governing the biosynthesis of fumonisins. Simultaneously, changes in biosynthesis of fumonisins were observed [29,30].

Furthermore, proteomic analyses brought us the knowledge about some of the proteins induced in strains treated with asparagus extract [31]. Obtained information is not sufficient to reveal the molecular mechanisms of *Fusarium* reaction under biotic stress conditions, therefore, the aim of this study was to investigate the effect of bioactive compounds obtained by fractionation of asparagus extract on fumonisins production, *FUM1* expression and dry biomass changes in the *Fusarium proliferatum* strain. Different solvents were tested (methanol, ethanol, methanol:water 1:1 and water) for extraction of the bioactive compounds from asparagus extract. In addition, to investigate the effectiveness of extracting the free and bound bioactive compounds, the alkaline and acidic hydrolysis extraction were used for the substances bound to the cell walls.

## 2. Results

### 2.1. Fumonisins

Fumonisins were detected both in controls as well as in the samples after extract and extract fractions treatment. Total fumonisin content reflects similar trend to that represented by individual FB_1_, FB_2_ and FB_3_ (Table 1). The FBs concentrations were higher in the first three time points and much lower at the end of the experiment. In the first and second (2 h) time point, total fumonisins content was higher for all asparagus extract fractions compared to the control with methanol. High FBs content was maintained only in *F. proliferatum* cultures treated with I and II extract fractions after 24 h since the beginning of the treatment .

**Figure 1:**
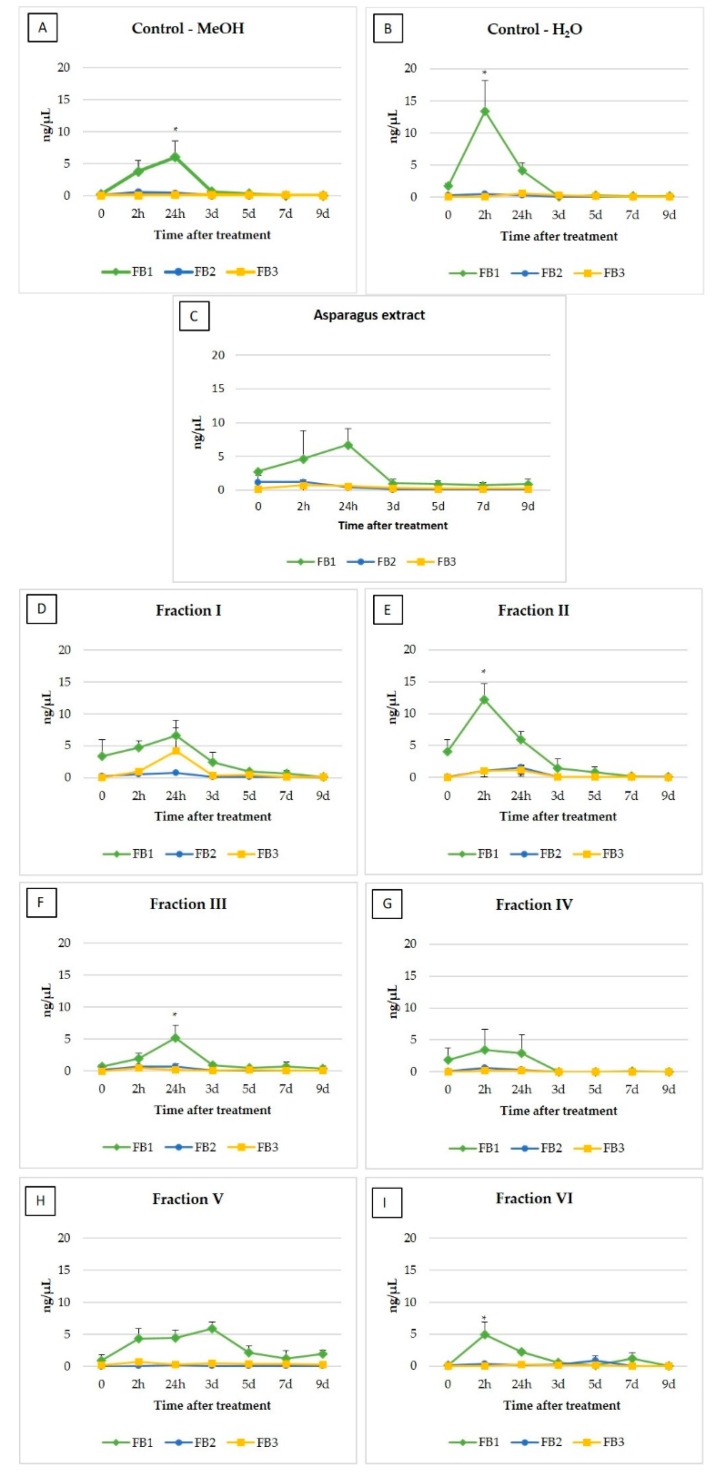
Changes of fumonisin B_1_, B_2_ and B_3_ concentrations in liquid medium during *F. proliferatum* cultures (* *p* < 0.05). (**A**)- H_2_O control, (**B**)- MeOH control, (**C**)- asparagus extract, (**D**–**I**)- asparagus extract fractions.

The average of maximum values measured in tested samples were 15.30, 1.53 and 4.24 ng/μL, whereas the average minimums were 0.04, 0.01 and 0.03 ng/μL for FB_1_, FB_2_ and FB_3_, respectively. Changes in fumonisin B_1_, B_2_ and B_3_ production occurring over the time of the experiment are shown in Figure 1. In almost all experimental variants, FBs showed similar distribution-fumonisin B_1_ level increased reaching the maximum in the 2nd or 24th hour after the treatment, then slightly decreased until the second day to reach the level just above the limit of detection. The cultures with fraction V or VI applied as a stressor, but in the case of fraction VI, this tendency was less visible. Fraction V maintained FB_1_ biosynthesis throughout the experiment but the production declined in the third day after the treatment. Generally, other analogues of fumonisins were produced at lower levels or were not detected, which particularly applies to FB_3_ for which no uniform trend was observed, except for fraction I. The only visible change was slight induction of fumonisin FB_2_ biosynthesis 24 h after the addition of fractions II and VI. Most of the asparagus extract fractions caused lower induction of fumonisins biosynthesis compared to asparagus extract. The differences in the activity of individual fractions may result from the difference in solvent polarities used, which also play a key role in increasing the solubility of bioactive compounds [32]. ANOVA showed an influence of asparagus extract as insignificant (*p > 0.05*).

Many bioactive compounds can be found in different plants, including phenolics, carotenoids, anthocyanins, vitamins and tocopherols. Asparagus is a rich source of bioactive compounds, containing phenolic compounds, flavonoids, alkaloids, tannins, sterols, amino acids, vitamins, saponins and fructans [33,34]. Most of the above-described groups of complex compounds were identified in obtained fractions but their intensities depended on polarity of the solvent used (data not shown). Adding a hydrolysis step to the fractionation process resulted in an increased number of free moieties compared to the fractions without a hydrolysis step. Since the obtained fractions were re-suspended in methanol, it was decided to apply two controls—first with the addition of water and second with methanol. The fumonisins level was about three times lower in the control with methanol compared to the water control, and differed significantly (*p < 0.05*). The distribution of FBs in time was similar, and for this reason, all results were referred to the control with methanol. Two hours after the beginning of the treatment, a higher level of FB_1_ was observed in the cases of fractions II, IV and VI, but only results for the fractions II and VI were statistically significant (*p < 0.05*) (Figure 1). Fraction II caused the highest induction of FB_1_, which reached the mean concentration of 12.20 ng/μL. In turn, after 24 h, higher values were observed only after fraction III application and the difference was also statistically significant (*p < 0.05*) from the control with methanol.

### 2.2. Analysis of FUM1 Gene Expression

**Figure 2:**
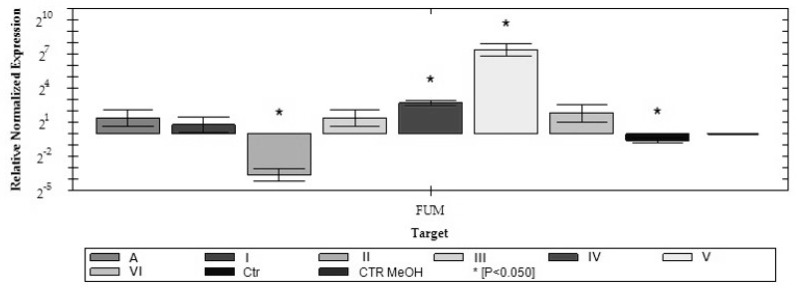
Normalized expression of the *FUM1* in *F. proliferatum* strain culture supplemented with asparagus extract fractions after 9th day of treatment (*p < 0.05).

Despite the low concentrations of fumonisins in the liquid medium at the last day of cultivation, we assumed that the expression of the key enzyme from fumonisin biosynthetic pathway-*FUM1*, encoding polyketide synthase could be altered during the culture. Knowing that the expression still occurs would improve the understanding of fumonisin accumulation, as well as clarify the influence of the whole extract and extract fractions on fumonisin biosynthesis. Relative normalized gene expression analysis was performed and is shown in Figure 2. The normalized *FUM1* gene expression of the KF 3360 *F. proliferatum* strain at 14th day of cultivation in response to whole water asparagus extract and different extract fractions was measured. The levels of the *FUM1* transcript varied relating to extract fractions used. The fraction V induced the highest expression level and the fraction IV was the second most effective inducer compared to the control (with methanol). The results revealed that fraction II inhibited the expression of the *FUM1* gene, which suggested that some extract compounds might have a certain effect on secondary metabolism gene expression. Surprisingly, the expression of the *FUM1* gene after whole asparagus extract supplementation did not change significantly *(p > 0.05).* ANOVA showed that fractions II, IV and V had statistically significant effects on the *FUM1* gene expression *(p < 0.05).*

### 2.3. Effect of Extract Fraction on Fungal Biomass

**Figure 3:**
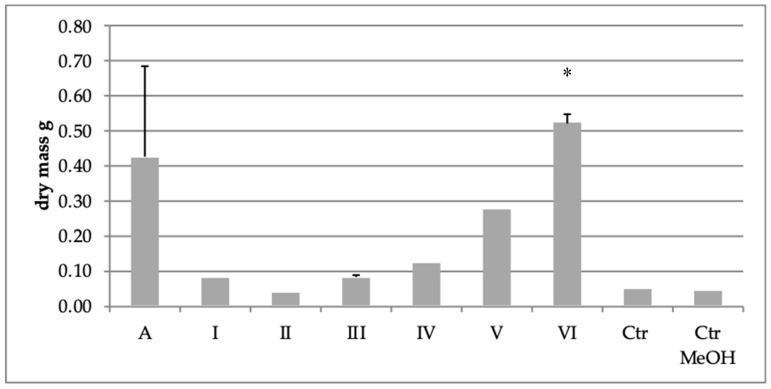
Changes of *F. proliferatum* dry biomass after treatment with crude extract (A), six asparagus extract fractions (I–IV), water and methanol controls (Ctr and Ctr MeOH) (*p < 0.05).

An asparagus-derived strain yielded between 40 and 520 mg of dry weight of mycelia at the end of cultivation (Figure 3). Fungal biomass amounts showed changes under experimental conditions. In all samples, increase in dry weight of mycelia was observed in comparison to the control. Among all extract fractions, fraction VI had the highest significant effect (*p < 0.05*) on dry weight of the mycelium after 14 days of cultivation, about 13-fold increase compared to the water control. Supplementation with methanol had a slight effect on biomass amount. Noteworthy, the asparagus extract induced about a 10-fold increase of fungal biomass compared to the control, however, the difference observed was not statistically significant *(p > 0.05).*

## 3. Discussion

Changes caused by *Fusarium*-produced fumonisin B_1_ in plants are generally well-known, comparing to the limited knowledge about the changes occurring in pathogen during this interaction. It is clear that fumonisin B_1_ plays a crucial role in activation of plant immunity; therefore, the mycotoxin was selected as an indicator of the intensity of plant-pathogen interaction in the present study. The interaction between a pathogen and a host is a continuous battle in which each action is followed by a reaction. It has been proven that FB_1_ causes an FB_1_-induced response in plants. In our studies, the increasing trend in the production of this mycotoxin in the first phase of infection might be a sign of FB_1_-induced response or HR, which are the early stage responses. However, research conducted by Waśkiewicz et al. [23] in asparagus showed that FB_1_ causes the increase in salicylic acid concentration, and, thereby, also a systemic acquired resistance (SAR) is induced, which might indicate that FB_1_ production does not lose its relevance during the course of the infection. Another explanation may be the plant’s secretion of a specific compound (or compounds) against the pathogen at the first contact, which might be equivalent to fungal FB_1_. Some compounds with antifungal activities were previously reported, including phytoalexins, for instance, pisatin from *Pisum sativum* L. against *Fusarium solani,* resveratrol from grapes against yeast or camalexin from *Arabidopsis thaliana* against *Alternaria brassiciola* [26,27,28].

Few studies were conducted analyzing the effect of plant extracts against *Fusarium* species and fumonisin biosynthesis. Research conducted by Suárez-Jiménez et al. [35] showed that methanolic extracts of *Larrea tridentata*, *Baccharis glutinosa* and *Ambrosia confertiflora* induced FB_1_ biosynthesis in *Fusarium verticillioides*, which is a close relative of *F. proliferatum* and also belongs to the *Liseola* section. Similar research was conducted by Thippeswamy et al. [36]. This research group tested aqueous extracts from 48 medicinal plants against *F. proliferatum* as well as the inhibition of fumonisin B_1_ production. *Asparagus racemosus* was among the plant species examined and it had no inhibitory properties on FB_1_ biosynthesis [36]. Some studies on the application of plant extracts against other *Fusarium* species, like *F. oxysporum*, *F. graminearum* or *F. sporotrichioides*, were also performed [37,38,39]. In our previous study, 16 isolates of *F. proliferatum* obtained from different host-plants have been exposed to the extracts obtained from maize, garlic, pea, pineapple and asparagus [30]. In most cases, application of the extract caused an increase in fumonisin B_1_ production and the distribution of its biosynthesis was comparable to the trend presented in the present study. The addition of the extract or extract fraction immediately caused a sudden increase in the amount of FB_1_. In conclusion, the results obtained by various research groups might suggest that the plants’ compound or group of compounds produced against *Fusarium* are not species-specific.

Fractionation of plant extracts is a complex way to select an antifungal agent. Correctly selected parameters of the extraction process allow to obtain from these extracts the maximum number of compounds with the highest biological activity [40]. One of the most important factors affecting the efficiency of bioactive compounds extraction from the plant samples is the extraction solvent. Due to the variety of bioactive compounds present in plant samples and their solubility depending on the polarity of the solvent, selection of an optimal solvent for a particular plant is quite difficult [41]. Many solvents of different polarities including methanol, ethanol, acetone, diethyl ether, water or their mixtures, should be used for extracting bioactive compounds from plants, applying the rule-a solvent will properly dissolve the solute of similar polarity [42]. Da Cruz-Silva et al. [43] conducted research on *Randia nitida* extracts and their fractions concerning their influence on *Colletotrichum truncatum*, *Rhizoctonia solani* and *Sclerotinia sclerotiorum* growth. The leaves extract was subjected to fractionation using methanol, *n*-hexane, dichloromethane and ethyl acetate, proving that each of them differed in terms of content of compound groups as well as their quantities. For example, methanolic fraction contained phenolic compounds, tannins, flavonoids, coumarins, triterpenes, steroids and alkaloids, while *n*-hexane fraction contained only triterpenes and steroids. Methanolic and ethyl acetate fractions contained the same groups of compounds, but in ethyl acetate fraction, lower amounts of tannins, triterpenes and steroids were detected [43]. Sales et al. [44] applied the extracts, along with acetate, butanol, dichloromethane, *n*-hexane and ethanolic fractions of extracts of 60 plants against two pineapple pathogens: *Fusarium guttiforme* and *Chalara paradoxa.* Extract and/or fractions obtained from 16 species were effectively inhibiting *F. guttiforme* [44]. In turn, Pizollito et al. [45] conducted the experiment with the use of fractions obtained from peanut skins. In this research, ethanolic extract (70:30, v:v) was fractionated with *n*-hexane, ethyl acetate and water, which resulted in the formation of three fractions: yellow, purple and brown, respectively. Yellow fraction showed the highest activity against *F. verticillioides* and fumonisin B_1_ accumulation [45]. Based on that, we might conclude that the fractions obtained in our experiments represent a wide cross-section of chemical compounds. The highest induction of FB_1_ biosynthesis was observed during the treatment of *F. proliferatum* culture with extract fraction I, which is an aqueous fraction, and fraction II-diethyl ether fraction with 2 M NaOH. Interestingly, the distribution of fumonisins in time was different in samples treated with fraction V (ethanolic fraction). On the contrary to other fractions, the content of fumonisins during the whole experiment did not reach the zero level, which suggests that the V fraction might contain compounds that stimulate continuous biosynthesis of this mycotoxin.

To further analyze the relationship between the levels of fumonisins produced and the composition of the extract fractions used, we measured the relative expression of the *FUM1* gene. Earlier studies have shown a linear relationship between the *FUM1* transcript and fumonisin production in vitro [46,47,48,49,50]. Although, in the present study, it was difficult to find similar correlation between *FUM1* transcript and fumonisin level at the 8th day of culture after fraction treatment. Our previous study has shown that the *FUM1* transcript levels were highly increased after exposure of the fungus to asparagus extracts, although previous findings suggested that in vitro expression of *FUM* genes in *F. proliferatum* is relatively stable and not depending on the culture conditions [30,47].

*F. proliferatum* supplemented with extract fraction V produced the highest amounts of fumonisins at the 9th day, showing the highest *FUM1* expression due to higher concentration of stimulating bioactive compounds. However, in the case of culture enriched with extract fraction IV, fumonisins were not detected at the 9th day, while the *FUM1* expression was still present. Medina et al. [51] and Battilanis et al. [52] suggested that the presence of a particular mycotoxin biosynthetic gene is not the only condition that has to be fulfilled by the pathogen to produce fumonisin. Moreover, previous studies described that the analysis of expression of the genes involved in fumonisin biosynthesis does not fully explain the regulation of their biosynthesis, however, it allows to better understand the changes in the pathogen’s physiology [30,46,53,54]. Notably, *FUM1* was down-regulated during increased FB_1_ production in the culture supplemented with extract fraction II. The mechanisms responsible for that might be independent of the activity of the *FUM* gene cluster. On the other hand, low fumonisin level in the medium supplemented with extract fractions I and III may result from the low activity of other genes from the FB biosynthetic cluster, which are responsible for processing of the pre-fumonisin compound [30,55,56]. It was suggested that the fungus protects the interior and exterior of its organism from harmful influence of mycotoxins by storing them inside the vacuoles and releasing it in the presence of stress factors [30]. Additionally, fumonisins can be transformed into different types, such as FAs, FCs or FPs by still unidentified enzymatic mechanisms [30,57].

Recently, some environmental and abiotic factors were reported to enhance the mycelia growth of *F. proliferatum*, including light, pH and nutrient level, as well as host plant extracts [29,30,53,54,55,56,57,58]. Our results indicate that extract fractions acted differently and generally increased fungal biomass of *F. proliferatum* after 14 days of culture, and extract fraction VI induced the highest amounts of FBs compared to the control. These results suggest the role played by the substrate in fungal growth rate-the fraction VI is richer in nutrients than fractions I–V. A possible explanation of why the biomass was more affected by the fraction VI might be due to the sudden delivery of the rich and easily accessible carbon source [29]. On the other hand, the slight decrease in fungal biomass in the case of the extract fraction II may result from the presence of bioactive compounds with antifungal properties such as flavonoids [59]. It is remarkable that the highest total amount of fumonisins in culture supplemented with extract fraction II was observed simultaneously with low dry weight of *F. proliferatum*. This finding suggests that the stressful environment created by the fraction II, as well as physiological response to overcome these conditions, might induce the fumonisin production.

It is important to emphasize that previous studies on the *F. proliferatum* pathogen treated with the host plant extract corresponded to this study’s results. It has been demonstrated that asparagus extract induced fungal biomass production (about two-fold increase) [29]. A similar reaction was observed for pineapple or maize extracts [30]. In our study, fungal biomass treated with whole asparagus extract was found to be higher than the control group, still not being statistically significant. Moreover, the differences in fungal biomass amounts between the whole extract and its fractions might have resulted from synergistic action of all bioactive compounds present in the asparagus extract.

## 4. Conclusions

Asparagus is a valuable crop with many beneficial features. It is often attacked by fungal pathogens and *Fusarium proliferatum* is one of the most dangerous ones. The species produces fumonisins and it has been found that supplementing the culture with the host plant extract changes the metabolism of the pathogen and its mycotoxigenic potential. Here, we have proven that fractions of the asparagus extract obtained using different solvents differed in the effect observed. Ethanolic and methanolic fractions induced the highest fumonisin biosynthesis. In addition, *FUM1* gene expression changed when the extract fractions were applied to the culture. Further research is needed to separate and identify chemical compounds responsible for the changes in fumonisin and biomass production.

## 5. Materials and Methods

### 5.1. Fungal Strain and Culture Conditions

An asparagus-derived KF 3360 *Fusarium proliferatum* strain from the KF pathogenic fungi collection at the Institute of Plant Genetics, Polish Academy of Sciences, Poznań, Poland, was used for this study. Based on the phenotypic variation and genetic divergence, selection of the strains was performed during earlier studies [29,60]. The KF 3360 strain, originally isolated from white asparagus spear (*Asparagus officinalis* L.) was cultured on potato dextrose agar (PDA, Oxoid) medium at 25 °C for 7 days for inoculum preparation.

**Figure 4:**
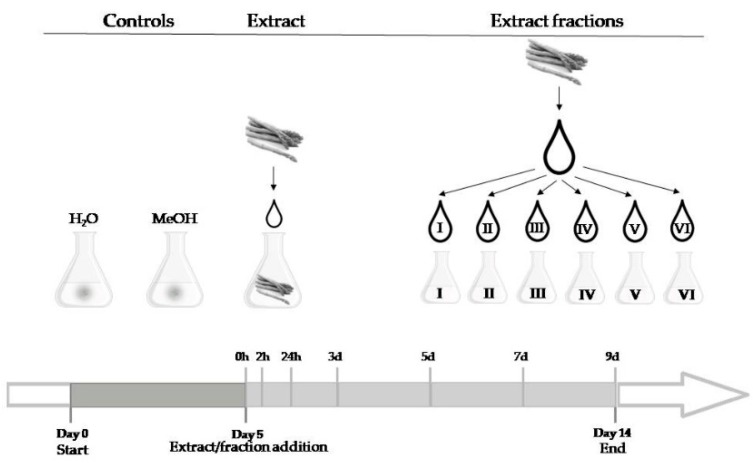
Scheme of the experimental design.

The fungus was cultured in vitro in 100 mL flasks containing 40–49 mL of a fumonisin-inducing liquid medium (25 °C without shaking at 12 h photoperiod) [29,46]. The medium contained: malt extract 0.5 g/L, yeast extract 1 g/L mycological peptone 1 g/L, KH_2_PO_4_ 1 g/L, MgSO_4_ × 7 H_2_O 0.3 g/L, KCl 0.3 g/L, ZnSO_4_ × 7 H_2_O 0.05 g/L, CuSO_4_ × 5 H_2_O 0.01 g/L and D-fructose 20 g/L. About 4 cm^2^ of mycelium harvested of the 7-day-old PDA plate cultures were used for the inoculation. At the 5th day of cultivation, the culture was supplemented with 10 mL of asparagus extract or 1 mL of I, II, III, IV, V, and VI fraction obtained according to the procedure described in Section 5.3. (Figure 4). A negative control was conducted to exclude the influence of methanol on fumonisins production and *FUM1* gene expression. The second negative control was the culture supplemented with water. Media of each culture were collected 2 h after the extract/fractions were added, and on 6th, 8th, 10th, 12th and 14th day of incubation, and subjected to the fumonisins quantification. Mycelia for dry weight measurement and *FUM1* gene expression analysis were harvested after 14 days of culturing, and immediately frozen in liquid nitrogen, and then freeze-dried. The concentrations of asparagus extracts, the time of application as well as culture conditions were optimized during earlier studies [30].

### 5.2. Extract Preparation

Extract of white asparagus spears was obtained according to Stępień et al. 2015 [29]. Fresh white asparagus spears without any symptoms of disease were frozen overnight at −80 °C, after that, completely defrosted asparagus spears were homogenized in a blender. Obtained pulp was centrifuged at 6000× *g* for 15 minutes. Extract was filtered through 0.20 μm membrane (Chromafil PET20/15 MS, Macherey-Nagel, Germany) and stored at –20 °C.

### 5.3. Fraction Preparation

Solvents used for the extraction of bioactive compounds from plants were chosen based on the different polarities. We tested six different solvents or solvent mixtures: water (I); methanol: water, 1:1, v:v (IV); ethanol (96%) (V); methanol (VI) in a 1:2 ratio (asparagus homogenate: solvent). Extraction was carried out in a water bath (60 °C, 4 h), shaken (24 h) and centrifuged. Then the supernatants were collected. For variants II and III, hydrolysis was performed in alkaline (2 M NaOH) and acidic (6 M HCl) medium, respectively. After 24 h of shaking, extraction was carried out 3 times with diethyl ether for both variants, and then, after combining the fractions, the solvent was evaporated to dryness. The dry residue was dissolved in a mixture of MeOH:H_2_O (1:1, v:v). Obtained supernatants were filtered through a 0.20 µm syringe filters (Chromafil PET20/15 MS, Macherey-Nagel, Germany) before use in microbiological tests.

### 5.4. Fumonisins Quantification

High purity mycotoxin standards (FB_1-3_, 50 µg/mL in acetonitrile: water, 1:1), LC/MS-grade organic solvents and other reagents were purchased from Sigma-Aldrich (Steinheim, Germany). The distilled water used for the studies was purified using a Milli-Q system (Millipore, Bedford, MA, USA). The analytical system consisted of the Aquity UPLC chromatograph (Waters, Manchester, MA, USA), coupled with an electrospray ionization triple quadrupole mass spectrometer (TQD) (Waters, Manchester, MA, USA). A Waters ACQUITY UPLC HSS T3 (100 × 2.1 mm/ID, with a particle size of 1.8 µm) (Waters, Manchester, MA, USA) was used for chromatographic separation, with a flow rate of 0.35 mL/min at room temperature. Mobile phase was composed of methanol (A) and water (B). Both phases contained 0.1% formic acid, phase B additionally contained 2 mM ammonium formate. The following gradient was used: from 1% to 95% A in 10 min, then 95% A for 2 min, and return to initial conditions in 2 min. The injection volume was 3 µL. Mass spectrometer was operated in the positive electrospray ionization mode (ESI). Ion source/desolvation temperature was 150/350 °C, respectively. Nebulizing gas (nitrogen) flow rate was 750 L/min, cone flow rate was 20 L/min. The collision-induced decomposition was performed using argon as the collision gas, with a collision energy of 14–22 eV. The compounds were quantitatively analyzed using multiple reaction monitoring. The analytes were identified by comparing the retention times and m/z values obtained by MS and MS2 with the mass spectra (722.4/352.4, 706.4/336.4 and 706.4/170.4 for FB_1_, FB_2_ and FB_3_, respectively) of the corresponding standards tested under the same conditions. Limit of detection for fumonisins was 0.1 ng/µL. All samples were analyzed in triplicate. For data processing, Empower^TM^ 1 software was used (Waters, Manchester, UK).

### 5.5. Expression Analysis of FUM1 by RT-qPCR

To analyze the expression of *FUM1* in *F. proliferatum* after extract fraction treatment, total RNA was extracted and purified from 30 mg of lyophilized mycelium sampled at the 14th day of culturing using the Universal RNA Purification Kit (EURx, Gdansk, Poland), followed by treatment with RNase-free DNase set (Qiagene, Hilden, USA). The total RNA concentration was quantified using a NanoDrop ND-1000 (Thermo Fisher Scientific, Waltham, MA, USA) and the integrity of RNA was evaluated on a 1% agarose gel (100 V/20 min). Then, 1 µg of total RNA was reverse-transcribed using a High Capacity cDNA Reverse Transcription Kit (Applied Biosystems, Foster City, CA, USA). Reactions were incubated at 25 °C for 10 min, followed by 37 °C for 120 min and 85 °C for 5 min using BioRad C1000 thermal cyclers. The resulting cDNA was used as template for RT-qPCR (SsoAdvanced Universal SYBR Green Supermix, Bio-Rad, Hercules, CA, USA), with *β*-tubulin as endogenous control to normalize differences in mRNA quantity due to differing amounts of total RNA. Three biological and two technical replicates of each sample, along with a negative control, were included in each assay. Primers used for *β*-tubulin and *FUM1* gene expression analysis were as follows: PQTUB-F2 ACATCCAGACAGCCCTTTGTG; PQTUB-R2 AGTTTCCGATGAAGGTCGAAGA [47] and F1_PRO_F CAACCGGAGAGAGCATTTGT; F1_PRO_R TCTTGGACAGAGGGGAGAAA [30]. Target sequences were amplified in a 5-μL reaction containing 2.5 µL of SsoAdvanced universal SYBR Green supermix, 500 nM of each primer for *FUM1* and 250 nM of each primer for *β*-tubulin and 2 µL of cDNA template (dilution 1:10). The PCR cycling conditions were: initial denaturation at 95 °C for 30 s, followed by 40 cycles of denaturation at 95 °C for 10 s and annealing at 60 °C for 30 s. The melting curve analysis from 65–95 °C with 0.5 °C increment (5 s per step), confirmed primer pairs specificity.

### 5.6. Statistical Analysis

One-way analysis of variance (ANOVA) followed by the Tukey test (HSD) (5% level of significance) was used to evaluate differences of FB_1_, FB_2_ and FB_3_ production between asparagus extract fractions during the period of incubation. The results were analyzed using the STATISTICA 13.1. Mean values (n = 3) and error standard of individual characteristics were calculated.

Target gene expression (*FUM1*) was determined using the 2^−ΔΔCt^ method [61]. All data were analyzed using CFX Maestro 1.1 software (Bio-Rad, Hercules, CA, USA). The differences of *FUM1* gene expression between samples treated with fractions were evaluated using the one-way analysis of variance (ANOVA) (5% level of significance). The expression was transformed to ln(x) to reduce the variability among the data. Baseline correction and threshold setting were performed using the automatic calculation in the CFX Maestro 1.1 software (Bio-Rad, Hercules, CA, USA).

## Figures and Tables

**Table 1:** Average values (ng/μL) and standard error of total fumonisins content in *F. proliferatum* cultures treated with asparagus extract fractions (*p < 0.05).

	0	2h	24h	3d	5d	7d	9d
Ctrl + H_2_O	1.97 ± 0.52	13.88 ± 4.38	4.88 ± 0.07	0.34 ± 0.07	0.33 ± 0.06	0.19 ± 0.04	0.13 ± 0.04
Ctrl + MeOH	0.29 ± 0.07	4.26 ± 1.19	6.48 ± 1.94	0.71 ± 0.18	0.37 ± 0.08	0.12 ± 0.02	0.08 ± 0.01
Extract	15.30 ± 6.29	6.67 ± 1.25	7.86 ± 2.09	1.08 ± 0.27	1.31 ± 0.24	0.85 ± 0.17	1.35 ± 0.26
Fraction I	3.54 ± 1.08	6.23 ± 1.33	11.56 ± 1.71	2.88 ± 0.72	1.48 ± 0.22	0.85 ± 0.16	0.26 ± 0.02
Fraction II	4.18 ± 1.32	14.34 ± 3.71	8.70 ± 1.55	1.67 ± 0.46	1.02 ± 0.05	0.31 ± 0.22	0.23 ± 0.04
Fraction III	0.88 ± 0.21	3.07 ± 0.44	6.07 ± 1.57	1.03 ± 0.30	0.72 ± 0.12	0.81 ± 0.22	0.49 ± 0.11
Fraction IV	1.98 ± 0.60	4.24 ± 1.02	3.37 ± 0.89	n.d.	n.d.	0.06 ± 0.02	n.d.
Fraction V	1.01 ± 0.28	5.16 ± 1.33	4.91 ± 1.42	6.38 ± 1.88	2.58 ± 0.00	1.69 ± 0.35	2.30 ± 0.61
Fraction VI	0.20 ± 0.03	5.26 ± 2.32	2.64 ± 0.68	0.82 ± 0.12	1.01 ± 0.24	1.19 ± 0.38	0.09 ± 0.02

n.d.—not detected.

## References

[1] Fuentes Alventosa J.M., Moreno Rojas J.M. (2015). Bioactive compounds in Asparagus and impact on storage and processing. Processing and impact on active components in food.

[2] Negi J.S., Singh P., Joshi G.P., Rawat M.S., Bisht V.K. (2010). Chemical consituents of *Asparagus*. Pharmacogn. Rev..

[3] Hafizur R.M., Kabir N., Chishti S. (2012). Asparagus officinalis extract controls blood glucose by improving insulin secretion and β-cell function in streptozotocin-induced type 2 diabetic rats. Br. J. Nutr..

[4] Shao Y., Chin C.-K., Ho C.-T., Ma W., Garrison S.A., Huang M.-T. (1996). Anti-tumor activity of the crude saponins obtained from *Asparagus*. Cancer Lett..

[5] Wang J., Liu Y., Zhao J., Zhang W., Pang X. (2013). Saponins extracted from by-product of *Asparagus officinalis L*. suppress tumour cell migration and invasion through targeting Rho GTPase signalling pathway. J. Sci. Food Agric..

[6] Shimoyamada M., Suzuki M., Maruyama M., Watanabe K. (1996). An Antifungal Saponin from White *Asparagus* (*Asparagus officinalis L*) Bottoms. J. Sci. Food Agric..

[7] Shimoyamada M., Suzuki M., Sonta H., Maruyama M., Okubo K. (1990). Antifungal Activity of the Saponin Fraction Obtained from *Asparagus officinalis L*. and Its Active Principle. Agric. Boil. Chem..

[8] Zhu X., Zhang W., Zhao J., Wang J., Qu W. (2010). Hypolipidaemic and hepatoprotective effects of ethanolic and aqueous extracts from *Asparagus officinalis L*. by-products in mice fed a high-fat diet. J. Sci. Food Agric..

[9] Zhu X., Zhang W., Pang X., Wang J., Zhao J., Qu W. (2011). Hypolipidemic Effect of n-Butanol Extract from *Asparagus officinalis L*. in Mice fed a High-fat Diet. Phytotherapy Res..

[10] Elmer W.H. (2001). The Economically Important Diseases of Asparagus in the United States. Plant Heal. Prog..

[11] Pontaroli A.C., Camadro E.L. (2001). Increasing resistance to Fusarium crown and root rot in *asparagus* by gametophyte selection. Euphytica.

[12] Stępień Ł., Waśkiewicz A., Urbaniak M. (2016). Wildly growing asparagus (*Asparagus officinalis* L.) hosts pathogenic *Fusarium* species and accumulates their mycotoxins. Microb. Ecol..

[13] Vujanovic V., Hamel C., Yergeau E., St-Arnaud M. (2006). Biodiversity and Biogeography of Fusarium Species from Northeastern North American Asparagus Fields Based on Microbiological and Molecular Approaches. Microb. Ecol..

[14] Elmer W.H. (2015). Management of Fusarium crown and root rot of asparagus. Crop. Prot..

[15] Lumsangkul C., Chiang H.-I., Lo N.-W., Fan Y.-K., Ju J.-C. (2019). Developmental Toxicity of Mycotoxin Fumonisin B1 in Animal Embryogenesis: An Overview. Toxins.

[16] Berkey R., Bendigeri D., Xiao S. (2012). Sphingolipids and Plant Defense/Disease: The “Death” Connection and Beyond. Front. Plant Sci..

[17] Merrill Jr. A. (2011). H. Sphingolipid and glycosphingolipid metabolic pathways in the era of sphingolipidomics. Chem. Rev..

[18] Merrill A.H., Sullards M.C., Wang E., Voss K.A., Riley R.T. (2001). Sphingolipid metabolism: Roles in signal transduction and disruption by fumonisins. Environ. Heal. Perspect..

[19] Zoeller M., Stingl N., Krischke M., Fekete A., Waller F., Berger S., Mueller M.J. (2012). Lipid Profiling of the Arabidopsis Hypersensitive Response Reveals Specific Lipid Peroxidation and Fragmentation Processes: Biogenesis of Pimelic and Azelaic Acid1[C][W]. Plant Physiol..

[20] Salehi F., Behboudi H., Kavoosi G., Ardestani S.K. (2018). Oxidative DNA damage induced by ROS-modulating agents with the ability to target DNA: A comparison of the biological characteristics of citrus pectin and apple pectin. Sci. Rep..

[21] Xing F., Li Z., Sun A., Xing D. (2013). Reactive oxygen species promote chloroplast dysfunction and salicylic acid accumulation in fumonisin B1-induced cell death. FEBS Lett..

[22] Stone J.M., Heard J.E., Asai T., Ausubel F.M. (2000). Simulation of Fungal-Mediated Cell Death by Fumonisin B1 and Selection of Fumonisin B1–Resistant (fbr) Arabidopsis Mutants. Plant Cell.

[23] Waśkiewicz A., Irzykowska L., Drzewiecka K., Bocianowski J., Dobosz B., Weber Z., Karolewski Z., Krzyminiewski R., Goliński P. (2013). Plant-pathogen interactions during infection process of asparagus with Fusarium spp.. Open Life Sci..

[24] Rocheleau H., Al-Harthi R., Ouellet T. (2019). Degradation of salicylic acid by Fusarium graminearum. Fungal Boil..

[25] Hao G., Naumann T.A., Vaughan M.M., McCormick S., Usgaard T., Kelly A., Ward T.J. (2019). Characterization of a Fusarium graminearum Salicylate Hydroxylase. Front. Microbiol..

[26] Pueppke S.G., VanEtten H.D. (1976). Accumulation of pisatin and three additional antifungal pterocarpans in Fusarium solani-infected tissues of Pisum sativum. Physiol. Plant Pathol..

[27] Pedras M.S.C., Minic Z., Abdoli A. (2014). The phytoalexin camalexin induces fundamental changes in the proteome of Alternaria brassicicola different from those caused by brassinin. Fungal Boil..

[28] Houillé B., Papon N., Boudesocque L., Bourdeaud E., Besseau S., Courdavault V., Enguehard-Gueiffier C., Delanoue G., Guérin L., Bouchara J.-P. (2014). Antifungal Activity of Resveratrol Derivatives against Candida Species. J. Nat. Prod..

[29] Stępień Ł., Waśkiewicz A., Wilman K. (2015). Host extract modulates metabolism and fumonisin biosynthesis by the plant-pathogenic fungus Fusarium proliferatum. Int. J. Food Microbiol..

[30] Górna K., Pawłowicz I., Waśkiewicz A., Stepien L. (2016). Fusarium proliferatum strains change fumonisin biosynthesis and accumulation when exposed to host plant extracts. Fungal Boil..

[31] Górna K., Perlikowski D., Kosmala A., Stępień Ł. (2017). Host extracts induce changes in the proteome of plant pathogen Fusarium proliferatum. Fungal Boil..

[32] Naima R., Oumam M., Hannache H., Sesbou A., Charrier B., Pizzi A., El Bouhtoury F.C. (2015). Comparison of the impact of different extraction methods on polyphenols yields and tannins extracted from Moroccan Acacia mollissima barks. Ind. Crop. Prod..

[33] Fuentes-Alventosa J., Jaramillo-Carmona S., Rodríguez-Gutiérrez G., Guillén-Bejarano R., Jiménez-Araujo A., Fernández-Bolaños J., Rodríguez-Arcos R. (2013). Preparation of bioactive extracts from asparagus by-product. Food Bioprod. Process..

[34] Lee J.W., Lee J.H., Yu I.H., Gorinstein S., Bae J.H., Ku Y.G. (2014). Bioactive Compounds, Antioxidant and Binding Activities and Spear Yield of Asparagus officinalis L.. Plant Foods Hum. Nutr..

[35] Suárez-Jiménez G.M., Cortez-Rocha M.O., Rosas-Burgos E.C., Burgos-Hernández A., Plascencia-Jatomea M., Cinco-Moroyoqui F.J. (2007). Antifungal activity of plant methanolic extracts against *Fusarium verticillioides* (Sacc.) Nirenb. and Fumonisin B_1_ Production. Rev Mex Fitopatol.

[36] Thippeswamy S., Umesh A.R. (2013). Effect of plant extracts on inhibition of *Fusarium verticillioides* growth and its toxin fumonisin B_1_ production. J Agri Sci Tech.

[37] Salhi N., Saghir S.A.M., Terzi V., Brahmi I., Ghedairi N., Bissati S. (2017). Antifungal Activity of Aqueous Extracts of Some Dominant Algerian Medicinal Plants. BioMed. Res. Int..

[38] Riaz T., Khan S., Javaid A. (2008). Antifungal activity of plant extracts against *Fusarium oxysporum-* the cause of corn-rot disease of Gladiolus. Mycopath.

[39] Rongai D., Pulcini P., Pesce B., Milano F. (2015). Antifungal activity of some botanical extracts on Fusarium oxysporum. Open Life Sci..

[40] Truong D.-H., Nguyen D.H., Ta N.T.A., Bui A.V., Do T.H., Nguyen H.C. (2019). Evaluation of the Use of Different Solvents for Phytochemical Constituents, Antioxidants, and In Vitro Anti-Inflammatory Activities of Severinia buxifolia. J. Food Qual..

[41] Mahdi-Pour B., Jothy S.L., Latha L.Y., Chen Y., Sasidharan S. (2012). Antioxidant activity of methanol extracts of different parts of *Lantana camara*. Asian Pac. J. Trop. Biomed..

[42] Wong P., Kitts D. (2006). Studies on the dual antioxidant and antibacterial properties of parsley (Petroselinum crispum) and cilantro (Coriandrum sativum) extracts. Food Chem..

[43] Da Cruz-Silva S.C.B., Santos K.S., Matias R., Bono J.A.M., Ludwig J. (2016). ANTIFUNGAL POTENTIAL OF EXTRACTS AND FRACTIONS OF Randia nitida LEAVES ON SOYBEAN PATHOGENS AND THEIR PHYTOCHEMISTRY. Rev. Caatinga.

[44] Sales M.D.C., Costa H.B., Fernandes P.M.B., Ventura J.A., Meira D.D. (2016). Antifungal activity of plant extracts with potential to control plant pathogens in pineapple. Asian Pac. J. Trop. Biomed..

[45] Pizzolitto R.P., Dambolena J.S., Zunino M.P., Larrauri M., Grosso N.R., Nepote V., Dalcero A.M., Zygadlo J.A. (2013). Activity of natural compounds from peanut skins on Fusarium verticillioides growth and fumonisin B1 production. Ind. Crop. Prod..

[46] López-Errasquín E., Vázquez C., Jiménez M., González-Jaén M.T., Escamilla M.J. (2007). Real-Time RT-PCR assay to quantify the expression of fum1 and fum19 genes from the Fumonisin-producing Fusarium verticillioides. J. Microbiol. Methods.

[47] Jurado M., Marín P., Callejas C., Moretti A., Vázquez C., González-Jaén M.T. (2010). Genetic variability and Fumonisin production by Fusarium proliferatum. Food Microbiol..

[48] Jurado M., Marín P., Magan N., González-Jaén M.T. (2008). Relationship between Solute and Matric Potential Stress, Temperature, Growth, and FUM1 Gene Expression in Two Fusarium verticillioides Strains from Spain. Appl. Environ. Microbiol..

[49] Rocha L.D.O., Reis G.M., Da Silva V.N., Braghini R., Teixeira M.M.G., Corrêa B. (2011). Molecular characterization and fumonisin production by Fusarium verticillioides isolated from corn grains of different geographic origins in Brazil. Int. J. Food Microbiol..

[50] Rocha L.O., Barroso V.M., Andrade L.J., Pereira G.H.A., Ferreira-Castro F.L., Duarte A.P., Michelotto M.D., Corrêa B. (2016). FUM Gene Expression Profile and Fumonisin Production by Fusarium verticillioides Inoculated in Bt and Non-Bt Maize. Front. Microbiol..

[51] Medina A., Schmidt-Heydt M., Cárdenas-Chávez D.L., Parra R., Geisen R., Magan N. (2013). Integrating toxin gene expression, growth and fumonisin B1 and B2 production by a strain of Fusarium verticillioides under different environmental factors. J. R. Soc. Interface.

[52] Battilani P., Formenti S., Ramponi C., Rossi V. (2011). Dynamic of water activity in maize hybrids is crucial for fumonisin contamination in kernels. J. Cereal Sci..

[53] Fanelli F., Schmidt-Heydt M., Haidukowski M., Geisen R., Logrieco A.F., Mulè G., Haidukowski E.M. (2012). Influence of light on growth, fumonisin biosynthesis and FUM1 gene expression by Fusarium proliferatum. Int. J. Food Microbiol..

[54] Lazzaro I., Susca A., Mulè G., Ritieni A., Ferracane R., Marocco A., Battilani P. (2012). Effects of temperature and water activity on FUM2 and FUM21 gene expression and fumonisin B production in Fusarium verticillioides. Eur. J. Plant Pathol..

[55] Proctor R.H., Brown D.W., Plattner R.D., Desjardins A.E. (2003). Co-expression of 15 contiguous genes delineates a fumonisin biosynthetic gene cluster in Gibberella moniliformis. Fungal Genet. Boil..

[56] Brown D.W., Cheung F., Proctor R.H., Butchko R.A., Zheng L., Lee Y., Utterback T., Smith S., Feldblyum T., Glenn A.E. (2005). Comparative analysis of 87,000 expressed sequence tags from the fumonisin-producing fungus Fusarium verticillioides. Fungal Genet. Boil..

[57] Tamura M., Mochizuki N., Nagatomi Y., Toriba A., Hayakawa K. (2014). Characterization of Fumonisin A-Series by High-Resolution Liquid Chromatography-Orbitrap Mass Spectrometry. Toxins.

[58] Kohut G., Ádám A.L., Fazekas B., Hornok L. (2009). N-starvation stress induced FUM gene expression and fumonisin production is mediated via the HOG-type MAPK pathway in Fusarium proliferatum. Int. J. Food Microbiol..

[59] Rosado-Álvarez C., Molinero-Ruiz L., Arcos R.R., Basallote-Ureba M. (2014). Antifungal activity of asparagus extracts against phytopathogenic Fusarium oxysporum. Sci. Hortic..

[60] Stępień Ł., Koczyk G., Waśkiewicz A. (2011). Genetic and phenotypic variation of *Fusarium proliferatum* isolates from different host species. J Appl Genet.

[61] Livak K.J., Schmittgen T.D. (2001). Analysis of relative gene expression data using real-time quantitative PCR and the 2(-Delta Delta C(T)) method. Methods.

